# Innovative Application of Three-Dimensional-Printed Breast Model-Aided Reduction Mammaplasty

**DOI:** 10.3389/fsurg.2022.890177

**Published:** 2022-06-09

**Authors:** Shaoheng Xiong, Bei E, Zhaoxiang Zhang, Jiezhang Tang, Xiangke Rong, Haibo Gong, Chenggang Yi

**Affiliations:** ^1^ Department of Plastic Surgery, Xijing Hospital, Fourth Military Medical University, Xi’an, China; ^2^ Department of Radiology, Xijing Hospital, Fourth Military Medical University, Xi’an, China; ^3^ The State Key Laboratory for Manufacturing Systems Engineering, Xi’an Jiaotong University, Xi’an, China; ^4^ Department of Plastic Surgery, The Second Affiliated Hospital, Zhejiang University School of Medicine, Hangzhou, China

**Keywords:** three-dimensional printing, macromastia, nipple-areolar complex, breast vascular model, reduction mammaplasty

## Abstract

Symptomatic macromastia places a severe physical and psychological burden on patients. Reduction mammaplasty is the primary treatment; however, conventional surgery may lead to postoperative nipple-areolar complex necrosis due to damage to the dominant supplying arteries. In this study, we designed and fabricated an innovative, three-dimensional-printed breast vascular model to provide surgical guidance for reduction mammaplasty. Preoperative computed tomography angiography scanning data of patients were collected. The data were then processed and reconstructed using the E3D digital medical modeling software (version 17.06); the reconstructions were then printed into a personalized model using stereolithography. The three-dimensional-printed breast vascular model was thus developed for individualized preoperative surgical design. This individualized model could be used to intuitively visualize the dominant supplying arteries’ spatial location in the breasts, thereby allowing effective surgical planning for reduction mammaplasty. The three-dimensional-printed breast vascular model can therefore provide an individualized preoperative design and patient education, avoid necrosis of the nipple-areolar complex, shorten operation duration, and ensure safe and effective surgery in patients.

## Introduction

Symptomatic macromastia places a heavy burden on patients and treatment is primarily via reduction mammaplasty (1). Necrosis of the nipple-areolar complex (NAC) is a severe complication of this surgery (2–5). Although recently, the necrosis rate of NAC has been reported to be decreasing with the improvement of surgery (6, 7), the risks of the larger amount of resection (>1,000 g/side) patients are still not negligible. Once necrosis occurs, it will be devastating for both surgeons and patients.

Adequate postoperative perfusion of the dominant supplying arteries of the nipple-areolar complex is thus vital for surgical success. Using computed tomography (CT) angiography, we previously revealed large individual variability in the dominant supplying arteries of the nipple-areolar complex in patients with macromastia (8). More than half of the patients had bilateral blood supply asymmetry; however, relying solely on radiographic imaging provides poor visualization of the three-dimensional (3D) spatial presentation. Converting images to a physical model by applying 3D printing technology may help solve the abovementioned problems in plastic surgery (9–11).

Here, we developed an innovative, 3D-printed breast vascular model to guide reduction mammaplasty, providing surgeons with accurate 3D spatial relationship data of between the dominant supplying arteries and adjacent tissues.

## Materials and Methods

This study was approved by the review board of Xijing Hospital of the Fourth Military Medical University. All patients provided informed consent to participate and for their data to be used. Inclusion criteria were bilateral symptomatic macromastia (volume >1,000 mL, measured by 3D scanning) with symptoms, including pain, restrictive respiratory effort, and psychological burden. Exclusion criteria were allergy to iodine contrast media, breast cancer, previous breast surgery, and ages <15 or >60 years.

### CT Angiography and Data Processing

Before scanning, plastic discs were pasted on both areolas to locate the bilateral nipple position for later reconstruction. CT angiography was performed using second-generation dual-source CT (SOMATOM Definition Flash; Siemens, Munich Germany). The raw CT imaging data were stored in DICOM format for thin-section reconstruction, with a reconstructed section thickness and interval of 0.625 mm each. For boundary segmentation and 3D reconstruction, the reconstructed images were imported into E3D software version 17.06 (Digital Medicine and Virtual Reality Research Center, Central South University, China). A senior radiologist marked the dominant source blood vessels of the breasts near the nipple-areolar complex and traced the original arteries in the reconstructed image. Engineers simplified the model structure according to our application requirements, retaining the dominant supply blood vessels, thorax, and partial bony structure, and used Cura 4.4.1 (Ultimaker, Utrecht, Netherlands) for subsequent 3D printing.

### 3D Printing Processes

**Figure 1:**
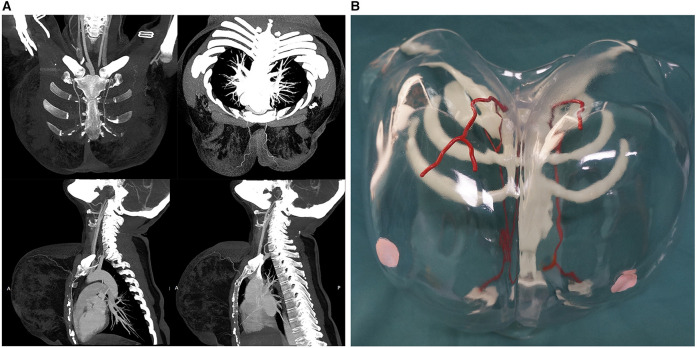
(**A**) Traditional computed tomography angiography. (**B**) Innovative three-dimensional (3D)-printed breast vascular model. The individualized 3D-printed breast vascular model includes the dominant supplying arteries (red), transparent chest with marked nipples (pink), sternum (white), and partial ribs (white).

The breast vascular model was made using photosensitive resin (ZR680; ZRapid Tech, Jiangsu, China); the flexural strength was 66–73 MPa, the percentage of breaking elongation was 10%–15% and materials were printed using a stereolithography apparatus (SL600, ZRapid Tech). First, the liquid, photosensitive resin was debubbled and loaded into the molding cylinder; the data were imported into the slicing software to slice and generate a G-code that the 3D printer would recognize. The photosensitive resin was solidified layer by layer under the control system using an ultraviolet (UV) lamp. After printing, the excess supporting structure was removed and sent to the posttreatment box for a UV lamp post-curing treatment. Finally, after polishing the surface, the desired 1:1 breast vascular model was prepared (Figure 1). The model was marked with different colors, representing the dominant supplying arteries of the nipple-areolar complex (red), the outline of the chest (transparent), sternum (white), and partial ribs (white).

### Surgical Design and Operation

**Figure 2:**
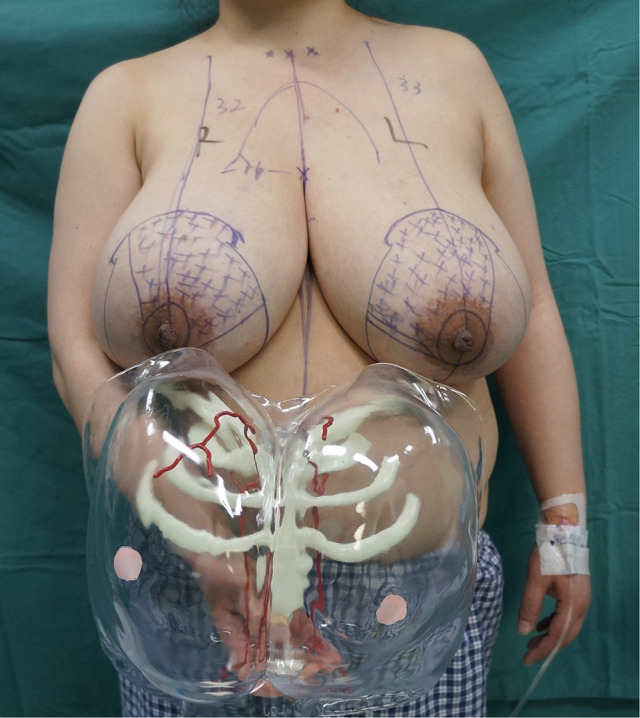
Personalized preoperative 3D-printed breast vascular model design.

**Figure 3:**
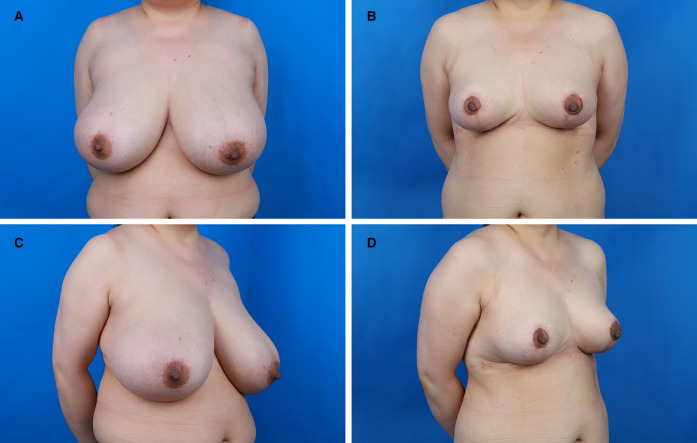
Preoperative and postoperative views of the patient. (**A**) Preoperative frontal view. (**B**) Postoperative frontal view. (**C**) Preoperative anterior-oblique view. (**D**) Postoperative anterior-oblique view.

The surgical type and resection sites depended on the location and course of the dominant supplying arteries of the nipple-areolar complex. Thus, before reduction mammaplasty, we marked the course of the arteries and important auxiliary lines on the surface of the breasts with the aid of the 3D-printed breast vascular model. These mainly determine the direction in which the breast preserves the glandular pedicles; for example, if the dominant arteries are derived from the internal thoracic artery and originate from the upper part of the breast, the direction of the glandular pedicles in the upper part of the inner section (Figure 2). If the preoperative bilateral breast volume is asymmetric, the larger breast requires multiple resections during the operation; thus, a 3D-printed breast vascular model could guide surgical excisions, avoiding damage to the dominant supplying arteries nipple-areolar complex (Figure 3).

## Discussion

The 3D-printed breast vascular model in this study was a 1:1 model designed according to the individual anatomical characteristics of each patient. It provides a practical approach to assist reduction mammaplasty, for which it can intuitively depict the 3D spatial relationships between the dominant supplying arteries of the nipple-areolar complex and surrounding tissues in the breast.

Although the safety of reduction mammaplasty has been greatly improved, it is still challenging for patients with a larger resection. Applying a 3D-printed breast vascular model could effectively avoid postoperative NAC necrosis. Besides, this would be a more valuable technique when reconstructing an area where the vascular supply has been disturbed by injury, radiation, or prior surgery.

The 3D-printed breast vascular model may contribute to good patient-physician communication, which helps to reduce postoperative medical disputes (12). Compared with conventional radiological images, the 3D-printed model may help explain the disease and improve patients’ understanding of the operation. Surgeons typically rely on personal experience and spatial reasoning to decide which part of the tissue should be removed. The clinical application of a 3D-printed model makes clinical surgery more visual and precise, helping to effectively narrow the gap between spatial imagination and physical models (13, 14). More importantly, this personalized model directly illustrates the precise 3D course of the dominant supplying arteries of the nipple-areolar complex, helping the surgeon plan this complex surgery and effectively reduce postoperative complications preoperatively.

Remarkably, this portable model can be taken directly into the operating room, allowing surgeons to easily visualize complex anatomical structures throughout the procedure (15). Typically, we carry out simultaneous bilateral reduction mammaplasty with the help of the model, which greatly shortens the operation duration and helps to reduce the risk of surgery (16). These 3D-printed models also represent a valuable educational resource for the surgical training of young doctors (17). Notably, the current quality of CT imaging can affect the accuracy of 3D-printed models; however, by upgrading the imaging equipment, high-resolution CT can further improve the accuracy of 3D-printed models. Besides, as we know that breasts are extremely mobile tissues, this would raise a concern about the changes between the anatomic locations of the vessels and breasts when standing and laying. Although we found that the relative position between the vessels and breasts is unchanged during the surgical operation, the deviations still need to be considered. To solve this, we are now designing and developing a special holder which could fix the position of the breasts during CT angiography with our engineers and radiologist. This holder could ensure the consistency of breast position during CT angiography and surgery planning, this part of the work is still in progress.

Another important significance of this study is that it can provide a promising paradigm for applying 3D printing in other fields of plastic surgery. This study adopts a multidisciplinary cooperation approach, which can make a better preoperative and more comprehensive evaluation and design with the help of the advantages of different disciplines, respectively, and can be extended to other operations with the help of 3D printed models. With the rapid development of 3D printing technology, its cost has been greatly reduced (18–20). The cost of the model used in this study is low and will not impose too much additional burden on patients (about 5% of the total surgical cost). Of course, in subsequent clinical trials, we will include the cost of 3D printing into the evaluation system to evaluate patients’ opinions on the cost of using 3D printing. The advantage of 3D technology is very obvious. In future research, we intend to apply it to breast reconstruction after injury, radiation, or tumor resection and expect to explore its more significant role in plastic surgery.

Of course, this study also has some limitations. The cost of CT angiography in China is not expensive (about 3% of the total surgical cost), but in other countries, this cost can not be ignored, which may cause an additional burden on patients. On the other hand, our radiologist has also evaluated the additional radiation caused by CT, which will not impact patients. Nevertheless, we are still looking for other more economical and less invasive alternatives, such as high-resolution ultrasound and color Doppler.

In conclusion, this is the first study to develop a 3D-printed breast vascular model to assist in reduction mammaplasty. This was found to be a simple, practical, and feasible method to improve patients’ understanding of the operation, avoid damage to the dominant arteries of the nipple-areolar complex during surgery, greatly shorten the operation duration, and reduce the risks of surgery.

## Data Availability

The original contributions presented in the study are included in the article/Supplementary Material, further inquiries can be directed to the corresponding author/s.
